# Practical clinical and radiological models to diagnose COVID-19 based on a multicentric teleradiological emergency chest CT cohort

**DOI:** 10.1038/s41598-021-88053-6

**Published:** 2021-04-26

**Authors:** Paul Schuster, Amandine Crombé, Hubert Nivet, Alice Berger, Laurent Pourriol, Nicolas Favard, Alban Chazot, Florian Alonzo-Lacroix, Emile Youssof, Alexandre Ben Cheikh, Julien Balique, Basile Porta, François Petitpierre, Grégoire Bouquet, Charles Mastier, Flavie Bratan, Jean-François Bergerot, Vivien Thomson, Nathan Banaste, Guillaume Gorincour

**Affiliations:** 1 Imadis Teleradiology, 48 Rue Quivogne, 69002 Lyon, France; 2 Centre Aquitain d’Imagerie, 64 rue de Canolle, 33000 Bordeaux, France; 3 Modelisation in Oncology (MOnc) Team, UMR 5251, INRIA Bordeaux-Sud-Ouest, CNRS, Université de Bordeaux, 33405 Talence, France; 4 Deeplink Medical, 22 rue Seguin, 69002 Lyon, France; 5 Norimagerie, Caluire et Cuire, France; 6 Imagerie Médicale du Mâconnais, Mâcon, France; 7 Ramsay Générale de Santé, Clinique de la Sauvegarde, Lyon, France; 8 Centre d’Imagerie Médicale Pourcel, Bergson, et de la clinique du Parc, Saint Etienne, France; 9 Ramsay Générale de Santé, Hôpital Privé Jean Mermoz, Lyon, France; 10 Service d’imagerie Diagnostique et Interventionnelle de l’adulte, Groupe Hospitalier Pellegrin, Place Amélie-Raba-Léon, 33076 Bordeaux cedex, France; 11 Department of Diagnostic and Interventional Imaging, Centre Hospitalier Saint-Joseph Saint-Luc, 20 Quai Claude Bernard, 69007 Lyon, France; 12 Department of Radiology, Centre Léon Bérard, Lyon, France; 13 Ramsay Générale de Santé, Clinique Convert, Bourg-en-Bresse, France; 14 Department of Radiology, Hopital Nord-Ouest, Villefranche-sur-Saône, France; 15 ELSAN, Clinique Bouchard, Marseille, France

**Keywords:** Diseases, Health care, Risk factors, Mathematics and computing

## Abstract

Our aim was to develop practical models built with simple clinical and radiological features to help diagnosing Coronavirus disease 2019 [COVID-19] in a real-life emergency cohort. To do so, 513 consecutive adult patients suspected of having COVID-19 from 15 emergency departments from 2020-03-13 to 2020-04-14 were included as long as chest CT-scans and real-time polymerase chain reaction (RT-PCR) results were available (244 [47.6%] with a positive RT-PCR). Immediately after their acquisition, the chest CTs were prospectively interpreted by on-call teleradiologists (OCTRs) and systematically reviewed within one week by another senior teleradiologist. Each OCTR reading was concluded using a 5-point scale: normal, non-infectious, infectious non-COVID-19, indeterminate and highly suspicious of COVID-19. The senior reading reported the lesions’ semiology, distribution, extent and differential diagnoses. After pre-filtering clinical and radiological features through univariate Chi-2, Fisher or Student t-tests (as appropriate), multivariate stepwise logistic regression (Step-LR) and classification tree (CART) models to predict a positive RT-PCR were trained on 412 patients, validated on an independent cohort of 101 patients and compared with the OCTR performances (295 and 71 with available clinical data, respectively) through area under the receiver operating characteristics curves (AUC). Regarding models elaborated on radiological variables alone, best performances were reached with the CART model (i.e., AUC = 0.92 [versus 0.88 for OCTR], sensitivity = 0.77, specificity = 0.94) while step-LR provided the highest AUC with clinical-radiological variables (AUC = 0.93 [versus 0.86 for OCTR], sensitivity = 0.82, specificity = 0.91). Hence, these two simple models, depending on the availability of clinical data, provided high performances to diagnose positive RT-PCR and could be used by any radiologist to support, modulate and communicate their conclusion in case of COVID-19 suspicion. Practically, using clinical and radiological variables (GGO, fever, presence of fibrotic bands, presence of diffuse lesions, predominant peripheral distribution) can accurately predict RT-PCR status.

## Introduction

Coronavirus disease 2019 (COVID-19) is a viral disease caused by severe acute respiratory syndrome coronavirus 2 (SARS-CoV-2). It was identified in Wuhan, China, in December 2019 and has rapidly spread worldwide^1^. As of February 13th, 2021, approximately 108 million patients have been reported worldwide, including 3,390,952 patients in France, with 80,404 deaths^2^. Real-time reverse transcription polymerase chain reaction (RT-PCR) is the gold standard for the acute diagnosis of SARS-CoV-2 in upper and lower respiratory specimens, despite possible inaccurate results (false negative and false positive)^3–6^. However, many hospitals have limited or delayed access to molecular testing. Conversely, chest CT is routinely performed at most institutions and can provide a result, or at least a diagnostic orientation, in less than an hour, which could help in patients’ triage.

Previous studies have highlighted typical COVID-19 patterns consisting of peripheral, multifocal ground-glass opacities (GGO), with a sensitivity of 60–98%^3,5,7,8^. Relying on these radiological features, the French Society of Radiology (SFR) has published educational webinars and a standardized report including a four-point scale to categorize the risk of COVID-19 on chest CT, namely: highly suspicious, compatible/indeterminate, not suspicious and normal (https://ebulletin.radiologie.fr/comptes-rendus-covid-19). Similar initiatives have been proposed by other radiological societies, such as the COVID-19 Reporting and Data System (CO-RADS) for the Dutch radiological society^9–11^. Overall, the specificity and sensitivity of these semi-quantitative scoring systems ranged from 0.45 (in asymptomatic patients) to 0.92, and from 0.66 to 0.94, respectively, when including the compatible/indeterminate category^9–14^.

In parallel, predictive models have been issued to facilitate and even automate the diagnosis of COVID-19 on chest CT with good performances and in an objective manner. Indeed, regarding deep-learning models, diagnostic performances (estimated with area under the receiver operating characteristics curves [AUC]) ranged from 0.70 to 0.95 in retrospective testing cohorts^15–20^. When detailed, sensitivity and specificity were 0.84–1 and 0.25–0.96, respectively. However, their implementation in practice requires either time or mathematical and computer sciences skills or graphics processing units. Alternatively, machine-learning models built on combinations of clinical, biological, radiological and even radiomics features have been developed^21,22^. Hence, Qin et al. have proposed a scoring system based on biological, clinical and radiological data with high performance (AUC = 0.91, sensitivity = 0.88, specificity = 0.92 in the independent validation cohort), but the added value to classical radiological assessment was not detailed^22^. Furthermore, some information required to run the model, notably a history of exposure or the leukocyte count, may not be systematically known by radiologists.

IMADIS Teleradiology is a French company dedicated to remote interpretation of emergency CT and MRI examinations. As of March 2020, IMADIS Teleradiology had partnerships with the emergency and radiological departments of 69 hospitals covering all French regions. During the coronavirus crisis, IMADIS has been widely involved in the diagnosis of COVID patients by remote interpretations of chest CT scans from partner centres. In order to rapidly homogenize the teleradiological managements of patients with a COVID-19 suspicion within our structure, a standardized and dedicated workflow and educational webinars were thus specifically developed, including the semi-quantitative scoring system derived from the SFR guidelines. Furthermore, through a systematic second reading of the teleradiological reports, the radiological semiology of each chest CT acquired in the workflow was collected.

Therefore, the first aim of our study was to elaborate and validate practical and simple classification models that could be used by any radiologist in any institution, without any mathematical background, based on easily available clinical and radiological features. Hence, in addition to semi-quantitative and subjective scoring systems, our models could provide a probability for a positive RT-PCR that could help modulating the traditional radiological assessment, improve the communication of the results to physicians and guide possible complementary diagnostic strategy in case of first negative swab. The second aim was to correlate the results of our models with those of the standardized conclusions of the IMADIS teleradiologists as given in a real-life emergency setting.

## Materials and methods

### Study design

This observational multicentric study was approved by the French Ethics Committee for the Research in Medical Imaging (CERIM) review board (IRB CRM-2007-107) according to good clinical practices and applicable laws and regulations. While the clinical and radiological data were prospectively collected, the study was designed retrospectively. Consequently, the written informed consent was waived due to the retrospective nature of the analysis. All data were anonymized before any analysis. All methods were performed in accordance with the relevant guidelines and regulations.

IMADIS Teleradiology is a medical company providing remote interpretation of emergency imaging examinations in dedicated on-call centres. As of March 2020, IMADIS Teleradiology had partnerships with the emergency and radiological departments of 69 hospitals covering all French regions.

**Figure 1:**
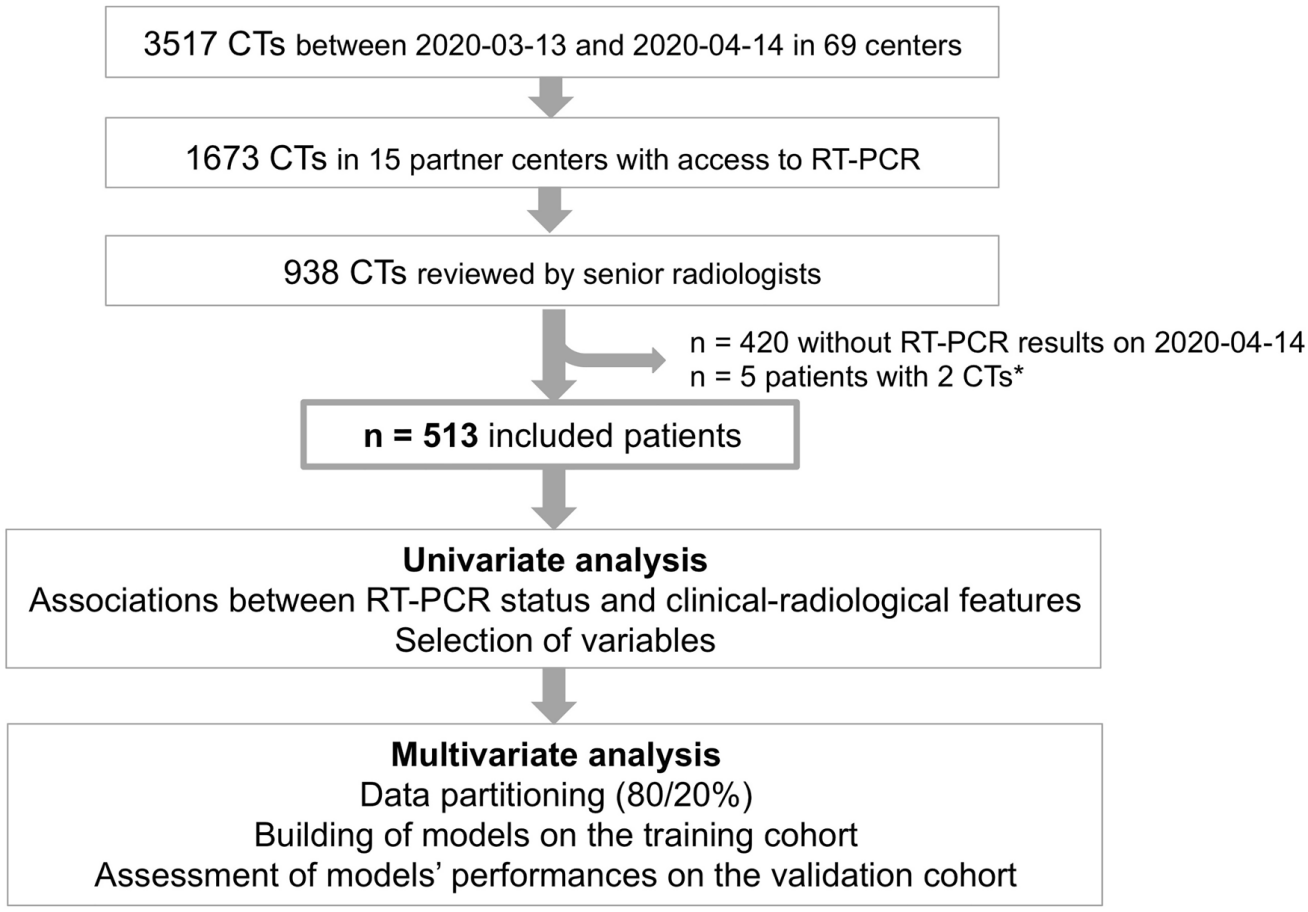
Flow-chart of the study.

Our study included all consecutive adult patients from 2020-03-13 to 2020-04-14 from 15/69 (21.7%) partner hospitals that regularly provided the RT-PCR results to IMADIS, as these patients fulfilled the following inclusion criteria: age above 18 years old, need for chest CT due to suspicion of COVID-19 according to a board-certified emergency physician, available chest CT, and available RT-PCR status (Fig. 1, Supplemental Data S1). We did not exclude patients because of their medical history.

### Chest CT acquisition

Chest CT examinations were performed by using 16- or 64-detector row CT scanners with a standardized non-contrast enhanced COVID-19 chest CT protocol for all hospitals. Depending on the emergency centers, the slice thickness ranged from 1 to 1.25 for the lung kernel, and from 2 to 2.5 mm for the mediastinal kernel. If pulmonary embolism was suspected, CT pulmonary angiographic protocol with bolus tracking intravenous iodine contrast agent administration at a rate of 3–4 mL/s was used instead (Omnipaque 350, GE Healthcare, Princeton, New Jersey; Iomeron 400, Bracco Diagnostics, Milan, Italy; and Ultravist 370, Bayer Healthcare, Berlin, Germany). In case of respiratory artifacts precluding the teleradiological interpretation, the acquisition was repeated, possibly on ventral decubitus.

### Teleradiological interpretation protocol

The panel of IMADIS teleradiologists consisted of 109 senior radiologists with at least 5 years of emergency imaging experience (mean length of practice, 7 years) and 55 junior radiologists (i.e., residents) with 3–5 years of emergency imaging experience (mean length of practice, 4 years). Teleradiologists were on-call in groups of at least two teleradiologists per night in each of the two interpretation centres (Bordeaux and Lyon, France). All radiological reports involving COVID-19 made by junior teleradiologists were validated by a senior teleradiologist working in the same interpretation center.

The IMADIS teleradiology interpretation protocol met the French recommendations for teleradiology practice^23^. The reports and requests with clinical data (filled by emergency physicians) for COVID-19 Chest CT image interpretation were sent from the client hospitals to the IMADIS Teleradiology interpretation centres by using teleradiology software (ITIS; Deeplink Medical, Lyon, France). The images were securely transferred over a virtual private network to a local picture archiving and communication system for interpretation available on each teleradiological workstation from the two interpretation centers (Carestream Health 12, Rochester, NY). Images were immediately interpreted by on-call teleradiologists (OCTRs) and the interpretation was subsequently transmitted to the emergency physician without any delay.

CT examinations were systematically reviewed within one week following each on-call session by another senior teleradiologist (n = 15; mean length of practice, 12.1 years; mean number of reviews, 34 CTs) who was not involved during the on-call duty period, blinded to the RT-PCR result and the first reader’s report. All senior radiologists had a 2-h-long e-learning session on CT-Chest findings in COVID-19, which became publicly available on April 7 (Web-based e-learning, developed by IMADIS Radiologists, Deeplink Medical, Lyon, France and RiseUp, Paris, France: https://covid19-formation.riseup.ai/) in addition to the educational webinars (https://ebulletin.radiologie.fr/cas-cliniques-covid-19) and guidelines (https://ebulletin.radiologie.fr/covid19?field_theme_covid_19_tid=613) provided by the SFR.

### Clinical data

Clinical information was provided by emergency physicians and collected through the teleradiology software in a standardized COVID-19 CT request form (ITIS; Deeplink Medical, Lyon, France), as follows: age; gender; active smoking; medical history, recent anti-inflammatory drugs intake; delay since onset of symptoms (categorized as: < 1 week, 1–2 weeks, ≥ 2 weeks); oxygen saturation (categorized as: ≥ 95%, 90–95% and < 90%); dyspnoea; fever (≥ 38 °C); cough; asthenia; headache; and ear, nose and throat symptoms.

The RT-PCRs were all performed on throat swab samples contemporary of the emergency room visit. Their results were retrospectively collected from the patients’ electronic medical records from each partner hospital.

### Radiological data

At the end of the report, the OCTR had to propose a conclusion adapted from the SFR classification (https://ebulletin.radiologie.fr/actualit%C3%A9s-covid-19/compte-rendu-tdm-thoracique-iv-0), as follows: (1) normal, (2) abnormalities inconsistent with pulmonary infection; (3) abnormalities consistent with a non-COVID-19 infection; (4) indeterminate/compatible abnormalities; and (5) findings strongly suspicious of COVID-19.

The 2nd reading assessed the following radiological features: (a) underlying pulmonary disease (categorized as: emphysema, lung cancer, interstitial lung disease, pleural lesions, or bronchiectasis); (b) GGO pattern (categorized as: rounded or non-rounded GGO); (c) consolidation pattern (categorized as: rounded or non-rounded consolidations and fibrotic bands [defined as thick, dense bands generally extending from a visceral pleural surface and possibly responsible for architectural distortions]); (d) predominant pattern (categorized as: GGO or consolidation); (e) distribution pattern of lesions (categorized as: peripheral predominant [defined as located within 3 cm of a costal pleural surface], central predominant, or mixed); (f) bilateral lesions; (g) diffuse lesions (i.e., five lobes involved); (h) basal predominant lesions; (i) pleural effusion (categorized as: uni- or bilateral); (j) adenomegaly (defined as lymph node with short axis > 10 mm); (k) bronchial wall thickening (when each bronchial wall approximately exceeds about 33% of the internal bronchial luminal diameter, which was further categorized as lobar/segmental or diffuse); (l) airway secretions; (m) tree-in-bud micronodules, and (n) pulmonary embolism. Images for each radiological feature can be found in Supplemental Data S2.

### Statistical analysis

Statistical analyses were performed using R (version 3.5.3, R Foundation for Statistical Computing, Vienna, Austria). All tests were two-tailed A p-value of less than 0.05 was deemed significant.

Univariate associations between clinical and radiological categorical variables and RT-PCR status were evaluated with Pearson Χ_2_ or Fisher exact tests, except for age, which was compared between the two groups with Student’s t-test (after assessing the normality of this numeric variable through Shapiro–Wilk test). Correlations between variables were evaluated with Spearman’s test in order to identify possibly redundant variables. For each significantly correlated pair of dummy variables extracted from the same initial multilevel categorical variable, the variable with the lowest p-value at univariate analysis was selected for the multivariable modelling.

Next, the study population was randomly partitioned into a training cohort (n = 412/513, ≈ 80%) and a validation cohort (n = 101/513, ≈ 20%), with a same prevalence of RT-PCR positivity (i.e. 196/412 [47.6%] and 48/101 [47.5%], respectively). We focused on two simple classifiers that do not require any computing interface to extract the probability for a positive RT-PCR, namely: classification and regression tree (CART, “rpart” R package) and stepwise backward-forward binary logistic regression (Step-LR—minimizing the Akaike information criterion, “MASS” R package). The models were built on the training cohort based on (i) either all dichotomized radiological variables or (ii) all dichotomized clinical + radiological variables—with a p-value < 0.05 at univariable analysis. The CART algorithm has one hyperparameter (i.e., a parameter that is set before the model building, while classical parameters are derived during the model building), named ‘complexity’, which controls the size of the tree and was selected following a cross-validation step in the training cohort as minimizing the classification error rate. Next, the tree was pruned following this optimal complexity hyperparameter. The minimal number of observations in the terminal node and the splitting criteria were set to 3 and the Gini index, respectively.

The performances of the CART-based and step-LR-based models were evaluated with AUC, i.e. by plotting the true positive rate (sensitivity) against the false positive rate (1—specificity) at various threshold settings and calculating the area under the curve. AUC was used to compare the models between themselves and to the prospective conclusions made by the OCTRs on the validation cohort. Accuracy (number of correctly classified patients divided by the total number of patients), sensitivity, specificity, negative predictive value (NPV) and positive predictive value (PPV) were estimated after dichotomizing predicted probabilities per a cut-off of 0.5. All results were given with a 95% confidence interval (95%CI). AUCs were compared using the pairwise Delong test (‘pROC’ R package).

Finally, we applied a decision curve analysis (DCA) to assess the clinical usefulness of the final models in the validation cohort. DCA consists of plotting the net benefit of applying the model for clinically reasonable risk thresholds compared with two alternative strategies: (i) to treat all patients as affected by COVID-19 or (ii) to treat none of the patients^24^. Herein, the net benefit of our models refers to the correct identification of patients with a positive or a negative RT-PCR, and the risk threshold can be seen as the harm-to-benefit ratio or the risk at which patients are indifferent about COVID-19^25^. Hence, a low risk threshold would correspond to patients who are particularly worried about the disease^26^.

## Results

### Study population

Table 1 summarizes the descriptive features of the study population. Overall, 513 patients were included, with a median age of 68.4 years (range 18–100) and 241/513 women (47%). Ninety-nine out of 513 (19.3%) of patients had a pre-existing lung chronic disease on chest CT. The prevalence of RT-PCR positivity was 244/513 (47.6%).

**Table 1:** Characteristics of the study population.

Characteristics	No. of patients
**Age (years old)**	
Mean	65.6 ± 18.8
Median (range)	68.4 (18.5–100.1)
**Gender**	
Female	241/513 (47%)
Male	272/513 (53%)
**Active smoking**	
No	359/412 (87.1%)
Yes	53/412 (12.9%)
**Anti-inflammatory drugs intake**	
No	395/418 (94.5%)
Yes	23/418 (5.5%)
**Significant medical history**	
No	151/478 (31.6%)
Cardiovascular risk factors and diseases	267/478 (55.9%)
Respiratory diseases	91/478 (19%)
Cancer	39/478 (8.2%)
Renal diseases	21/478 (4.4%)
Neurological diseases	44/478 (9.2%)
Liver diseases	4/478 (0.8%)
Immunodepression	19/478 (4%)
**Pre-existing lung chronic disease on chest CT**	
No	414/513 (80.7%)
Yes	99/513 (19.3%)
**RT-PCR status**	
Negative	269/513 (52.4%)
Positive	244/513 (47.6%)

*Note* Data refer to the number of patients with percentage in parentheses, except for age.

### Univariate analysis

The following clinical variables were associated with RT-PCR positivity (RT-PCR +): delay since onset of symptoms ≥ 1 week, oxygen saturation < 95%, oxygen saturation < 90%, presence of fever, cough, asthenia and myalgia *(*p = 0.04, 0.03, 0.005, < 0.001, 0.02, 0.001 and 0.008, respectively) (Table 2). On the contrary, the presence of a dyspnea, headache and ear, nose, throat symptoms did not correlate with the RT-PCR status (p = 0.16, 0.5 and 0.6, respectively).

**Table 2:** Univariate associations between clinical features and RT-PCR status.

Variables	RT-PCR − ^§^	RT-PCR + ^§^	*p* value
**Time since the onset of symptoms**			
< 1 week	148/240 (61.7%)	115/223 (51.6%)	0.02*
1–2 weeks	80/240 (33.3%)	102/223 (45.7%)	0.04*
> 2 weeks	12/240 (5%)	6/223 (2.7%)	0.3
**Dyspnea**			
No	56/269 (20.8%)	38/244 (15.6%)	0.16
Yes	213/269 (79.2%)	206/244 (84.4%)	
**Oxygen saturation**			
≥ 95%	77/195 (39.5%)	54/191 (28.3%)	0.006*
90–95%	82/195 (42.1%)	77/191 (40.3%)	0.03*
< 90%	36/195 (18.5%)	60/191 (31.4%)	0.005**
**Cough**			
No	85/269 (31.6%)	53/244 (21.7%)	0.02*
Yes	184/269 (68.4%)	191/244 (78.3%)	
**Fever (≥ 38 °C)**			
No	109/269 (40.5%)	34/244 (13.9%)	< 0.001***
Yes	160/269 (59.5%)	210/244 (86.1%)	
**Asthenia**			
No	125/269 (46.5%)	78/244 (32%)	0.001**
Yes	144/269 (53.5%)	166/244 (68%)	
**Myalgia**			
No	211/269 (78.4%)	165/244 (67.6%)	0.008*
Yes	58/269 (21.6%)	79/244 (32.4%)	
**Headache**			
No	228/269 (84.8%)	200/244 (82%)	0.5
Yes	41/269 (15.2%)	44/244 (18%)	
**Ear, nose throat symptoms**			
No	247/269 (91.8%)	228/244 (93.4%)	0.6
Yes	22/269 (8.2%)	16/244 (6.6%)	

P-values in bold correspond to significant associations between clinical features and RT-PCR status. Regarding variables with more than two levels, the p value in italics corresponds to the p-value considering all its levels. The p-values below are based on the Fisher or X_2_ test for this variable that was dichotomized according to the level of the line.

^§^Data refer to number of patients with percentage in parentheses.

*p < 0.05, **p < 0.005, ***p < 0.001 (corresponds to X_2_ or Fisher test).

The following radiological variables were positively associated with RT-PCR +: presence of GGO, non-rounded GGO, rounded GGO, presence of consolidation, non-rounded consolidation, fibrotic bands, intralobular reticulations, fibrosis, GGO predominant pattern, peripheral predominant location, bilateral lesions, diffuse lesions, basal predominant lesions, and low, moderate and high extent of abnormalities (all p-values < 0.001) (Table 3). The following radiological variables were negatively correlated with RT-PCR + : consolidation predominant pattern, central predominant location, mixed predominant location, airway secretion, bronchial wall thickening, either lobar/segmental or diffuse, and tree-in-bud micronodules (p = 0.02, 0.001, 0.002, < 0.001, < 0.001, < 0.001, < 0.001, < 0.001, respectively). On the contrary, the presence of rounded consolidation, pleural effusion and adenomegaly did not correlate with the RT-PCR status (p = 0.6, 0.2 and 0.3, respectively).

**Table 3:** Univariate associations between radiological features and RT-PCR status.

Variables	RT-PCR − ^§^	RT-PCR + ^§^	*p* value
**Presence of GGO**			
No	180/269 (66.9%)	15/244 (6.1%)	< 0.001***
Yes	89/269 (33.1%)	229/244 (93.9%)	
**Non-rounded GGO**			
Absent	197/269 (73.2%)	31/244 (12.7%)	< 0.001***
Present	72/269 (26.8%)	213/244 (87.3%)	
**Rounded GGO**			
Absent	238/269 (88.5%)	162/244 (66.4%)	< 0.001***
Present	31/269 (11.5%)	82/244 (33.6%)	
**Presence of consolidation**			
No	196/269 (72.9%)	92/244 (37.7%)	< 0.001***
Yes	73/269 (27.1%)	152/244 (62.3%)	
**Non-rounded consolidation**			
No	217/269 (80.7%)	172/244 (70.5%)	0.0097*
Yes	52/269 (19.3%)	72/244 (29.5%)	
**Rounded consolidation**			
No	246/269 (91.4%)	227/244 (93%)	0.6
Yes	23/269 (8.6%)	17/244 (7%)	
**Sub-pleural band**			
No	253/269 (94.1%)	142/244 (58.2%)	< 0.001***
Yes	16/269 (5.9%)	102/244 (41.8%)	
**Predominant pattern**			
None	150/269 (55.8%)	11/244 (4.5%)	< 0.001***
Consolidation	57/269 (21.2%)	32/244 (13.1%)	
GGO	62/269 (23%)	201/244 (82.4%)	
**Distribution pattern of lesions**			
None	133/269 (49.4%)	9/244 (3.7%)	< 0.001***
Peripheral predominant	59/269 (21.9%)	154/244 (63.1%)	
Central predominant	28/269 (10.4%)	7/244 (2.9%)	
Mixed	49/269 (18.2%)	74/244 (30.3%)	
**Bilateral lesions**			
No	170/269 (63.2%)	28/244 (11.5%)	< 0.001***
Yes	99/269 (36.8%)	216/244 (88.5%)	
**Diffuse lesions**			
No	231/269 (85.9%)	71/244 (29.1%)	< 0.001***
Yes	38/269 (14.1%)	173/244 (70.9%)	
**Basal predominant lesions**			
No	209/269 (77.7%)	145/244 (59.4%)	< 0.001***
Yes	60/269 (22.3%)	99/244 (40.6%)	
**Extent of lesions**			
None	134/269 (49.8%)	9/244 (3.7%)	< 0.001***
Low	79/269 (29.4%)	74/244 (30.3%)	< 0.001***
Moderate	46/269 (17.1%)	115/244 (47.1%)	< 0.001***
High	10/269 (3.7%)	46/244 (18.9%)	< 0.001***
**Intralobular reticulations**			
No	231/269 (85.9%)	103/244 (42.2%)	< 0.001***
Yes	38/269 (14.1%)	141/244 (57.8%)	
**Tree-in-bud micronodules**			
No	209/269 (77.7%)	230/244 (94.3%)	< 0.001***
Yes	60/269 (22.3%)	14/244 (5.7%)	
**Presence of bronchial wall thickening**			
No	194/269 (72.1%)	227/244 (93%)	< 0.001***
Yes	75/269 (27.9%)	17/244 (7%)	
**Distribution of bronchial abnormalities**			
None	194/269 (72.1%)	227/244 (93%)	< 0.001***
Lobar/segmental	29/269 (10.8%)	7/244 (2.9%)	< 0.001***
Diffuse	46/269 (17.1%)	10/244 (4.1%)	< 0.001***
**Airway secretion**			
No	208/269 (77.3%)	227/244 (93%)	< 0.001***
Yes	61/269 (22.7%)	17/244 (7%)	
**Fibrosis**			
No	253/269 (94.1%)	193/244 (79.1%)	< 0.001***
Yes	16/269 (5.9%)	51/244 (20.9%)	
**Pleural effusion**			
Absent	221/269 (82.2%)	215/244 (88.1%)	*0.2*
Yes, unilateral	18/269 (6.7%)	10/244 (4.1%)	0.3
Yes, bilateral	30/269 (11.2%)	19/244 (7.8%)	0.3
**Adenomegaly**			
No	248/269 (92.2%)	218/244 (89.3%)	0.3
Yes	21/269 (7.8%)	26/244 (10.7%)	

P-values in bold correspond to significant associations between clinical or radiological features and RT-PCR status. Regarding variables with more than two levels, the p value in italics corresponds to the p-value considering all its levels. The p-values below are based on the Fisher or X_2_ test for this variable that was dichotomized according to the level of the line.

*GGO* ground-glass opacities.

^§^Data refer to number of patients with percentage in parentheses.

*p < 0.05, **p < 0.005, ***p < 0.001 (corresponds to X_2_ or Fisher test).

### Multivariate models

**Figure 2:**
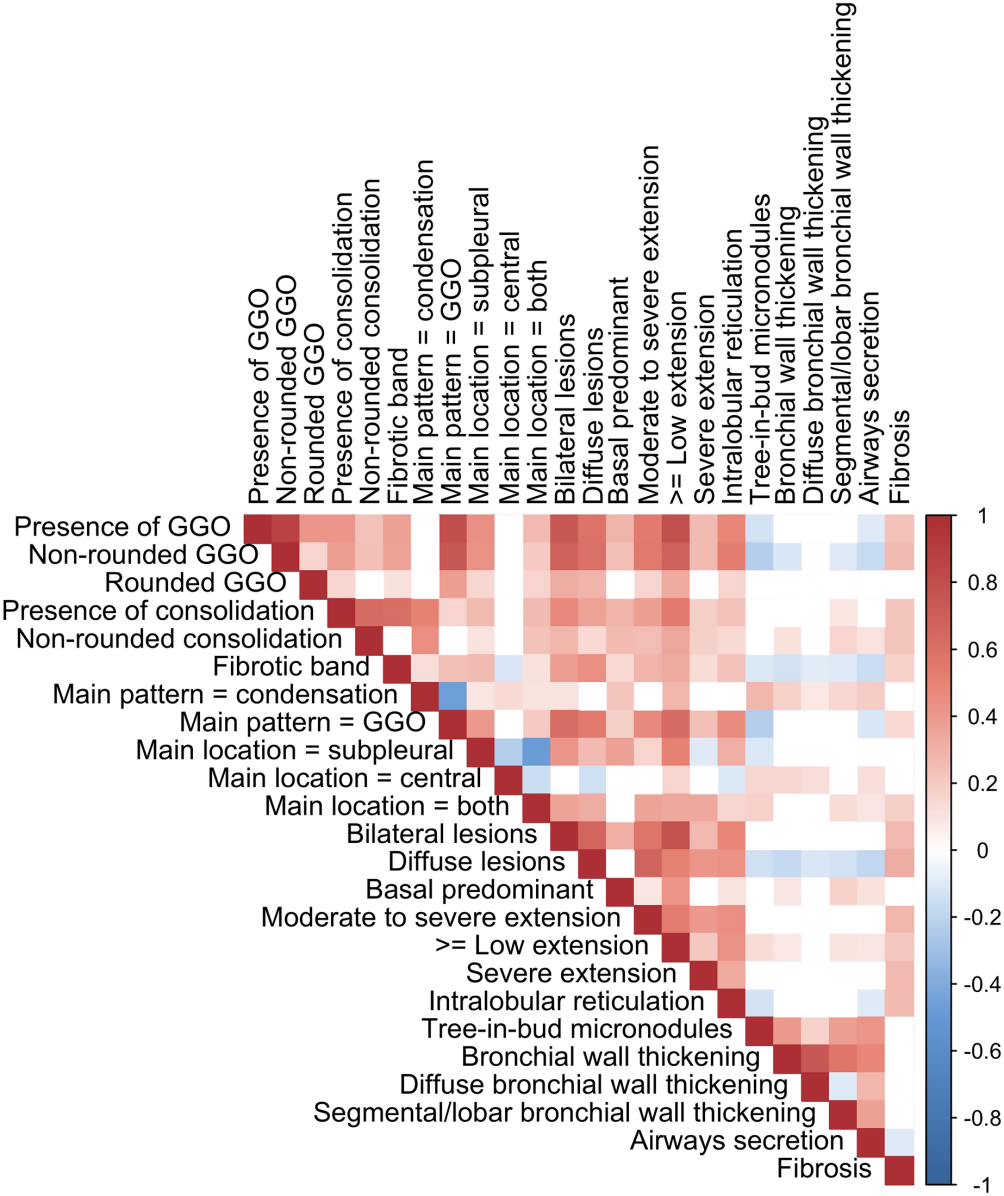
Correlation matrix of the 24 dichotomized radiological variables with a significant association with the RT-PCR status at univariate analysis. The colour-coding only corresponds to significant correlation (p < 0.05 according to Spearman test), from red (positive correlations) to blue (negative correlations). *GGO* ground-glass opacity.

The correlation matrix of the relevant dichotomized variables is shown in Fig. 2. Analysis of the correlations between similar explanatory variables enabled the selection of ‘presence of GGO’ (over ‘rounded GGO’ and ‘non-rounded GGO’), ‘peripheral predominant location’ (over ‘central predominant location’ and ‘mixed predominant location’), ‘fibrotic band consolidation’ (over ‘non-rounded consolidation’ and ‘presence of consolidation’), ‘moderate to severe extension’ (over ‘low to severe extension’ and ‘severe extension’), and ‘bronchial wall thickening’ (over ‘diffuse bronchial wall thickening’ and ‘focal/segmental bronchial wall thickening’). Thus, the total numbers of variables ultimately entered in the multivariate radiological and clinical-radiological models were set to 13 and 19 dichotomized variables, respectively.

**Figure 3:**
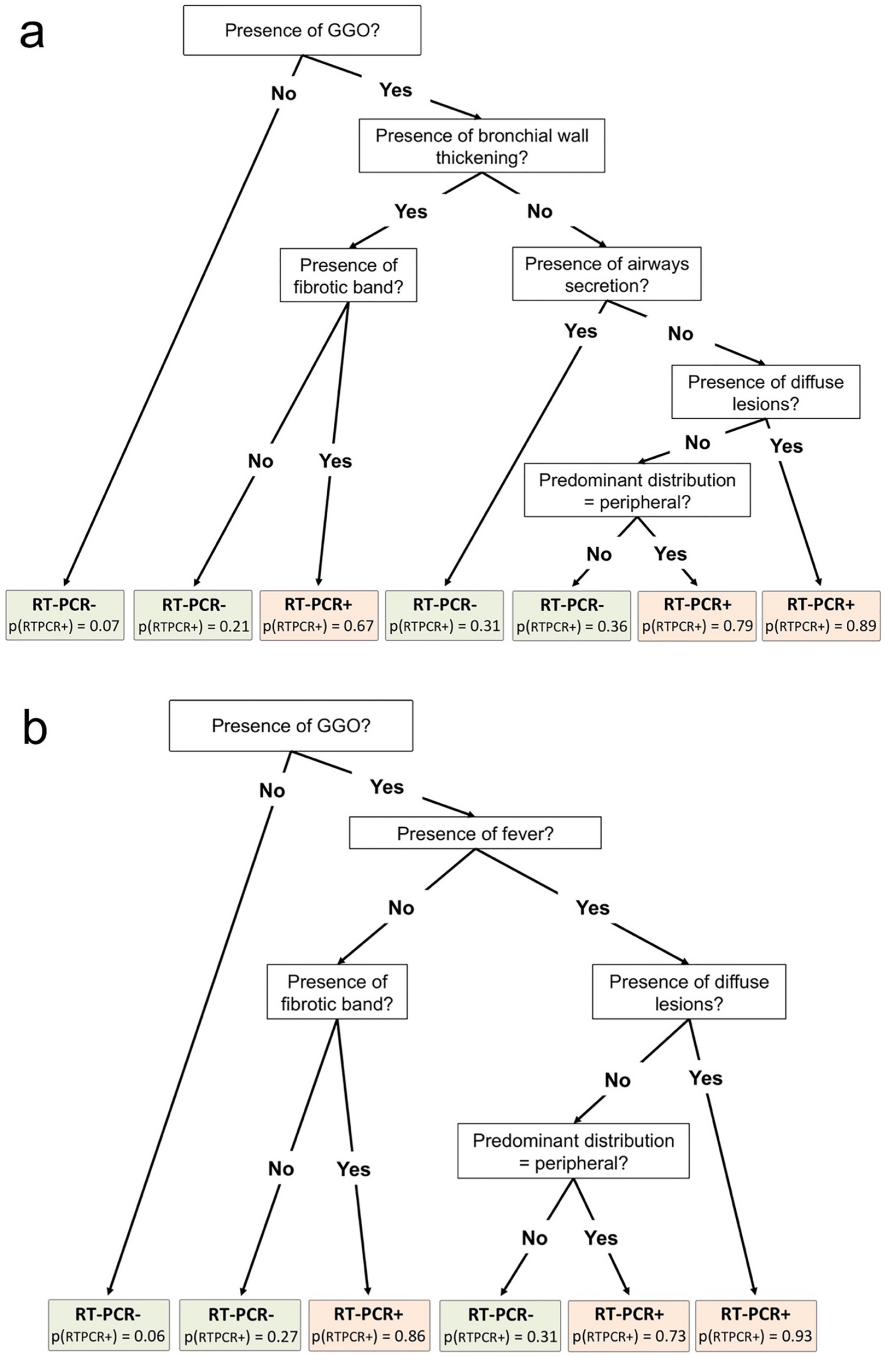
Final classification and regression tree (CART) models built with (**a**) radiological variables alone (with the highest performances in the validation cohort) and (**b**) clinical radiological variables. *GGO* ground-glass opacities, *p(RT-PCR +)* probability for a positive RT-PCR.

Figure 3 shows the final decision trees. Regarding the best tree relying on radiological variables, six nodes corresponding to six questions were used. Fewer nodes were needed with the clinical-radiological final tree model (i.e. five nodes) Hence, the probability for positive RT-PCR ranged from 0.07 to 0.89, and from 0.06 to 0.93, respectively.

Regarding the Step-LR models, Table 4 shows the matrices enabling the calculation of the probability for RT-PCR + depending on radiological and clinical-radiological variables. These models also enable to identify independent predictors for RT-PCR + , that-is-to-say: fever, fibrotic bands, a GGO predominant pattern, a peripheral predominant distribution, diffuse lesions, intralobular reticulations and absence of bronchial wall thickening (range of p-value =  < 0.001–0.03).

**Table 4:** Final multivariate stepwise binary logistic regression models elaborated with either radiological or clinical-radiological variables.

Variables^§^	Coefficients (βi)	P-value	Multivariate OR (95%CI)	Examples^§§^		
				A	B	C
**Radiological model**						
*0. (Intercept)*	− 2.758126351	–	0.06 (0.03–0.12)	1	1	1
1. Presence of GGO	1.089578460	0.04*	2.97 (1.03–8.56)	0	1	1
2. Fibrotic band	1.5310410593	< 0.001***	4.62 (2.11–10.77)	1	0	1
3. GGO predominant pattern	1.5366549953	< 0.001***	4.65 (1.96–11.45)	0	1	1
4. Subpleural predominant distribution	0.9053515712	0.004**	2.47 (1.33–4.63)	0	1	1
5. Diffuse lesions	0.8414110324	0.02*	2.32 (1.16–4.61)	0	0	1
6. Intralobular reticulations	0.7052466524	0.03*	2.02 (1.05–3.91)	0	0	1
7. Bronchial wall thickening	− 1.758954141	< 0.001***	0.17 (0.07–0.4)	1	0	0
Probablity for RT-PCR + ^§§^				4.8%	68.4%	97.9%
**Clinical-radiological model**						
*0. (Intercept)*	− 4.805582918	–	–	1	1	1
1. Fever	1.982642859	< 0.001***	7.26 (2.82–20.41)	1	1	1
2. Myalgia	0.899937144	0.08	2.46 (0.82–6.94)	1	0	0
3. Presence of GGO	1.181705996	0.08	3.26 (0.86–12.54)	1	1	0
4. Presence of fibrotic band	1.322814497	0.02*	3.75 (1.34–11.47)	1	0	1
5. GGO predominant pattern	1.504660153	0.008*	4.5 (1.52–14.24)	1	1	0
6. Peripheral predominant distribution	0.930711612	0.03*	2.54 (1.12–5.83)	1	1	0
7. Diffuse lesions	1.413720338	0.003**	4.11 (1.66–10.58)	1	0	0
8. Intralobular reticulations	1.028166435	0.02*	2.8 (1.2–6.66)	1	0	0
9. Bronchial wall thickening	− 1.716180719	0.002**	0.18 (0.06–0.51)	0	0	1
Probablity for RT-PCR + ^§§§^				99.6%	68.9%	3.9%

Significant results are highlighted in bold.

*95%CI* 95% confidence interval; *GGO* ground glass opacity; *OR* odds ratio.

^§^The variables correspond to those in the final model after the stepwise backward-forward selection.

^§§^Examples correspond to 6 distinct clinical cases. Each variable has 2 levels: “1” if the variable Xi is present (for instance fever), and “0” if the variable Xi is absent (for instance lack of fever).

^§§§^The probability for RT-PCR + are calculated as follows:

P(RT-PCR +) = $$ 1/1 + {\text{~exp}}[ - (\beta _{{0~}}  + \mathop \sum \nolimits_{{i = 1}}^{9} \beta _{i}  \times X_{i} )] $$.

*P < 0.05, **P < 0.005, ***P < 0.001.

### Performances of the models and conclusions by on-call teleradiologists

To evaluate the performances of the models, trained models were tested on the external validation cohort. Table 5 shows the results while results in the training cohort are given in Supplemental Data S3.

**Table 5:** Performances of the final, trained, multivariate models and on-call teleradiologists’ conclusions on the independent validation cohort.

Performance measures	Final models		OCTRs (validation cohort)
	Step-LR	CART	
**Radiological models (n = 101)**			
Accuracy^§^	0.83 (0.74–0.90)	**0.86 (0.78–0.92)**	0.81 (0.72–0.88)
OR^§^	26.3 (8.9–77.9)	**56.1 (14.6–215.3)**	26.9 (8.2–88.4)
Sensitivity^§^	**0.77 (0.63–0.88)**	**0.77 (0.63–0.88)**	0.69 (0.54–0.81)
Specificity^§^	0.89 (0.77–0.96)	**0.94 (0.84–0.99)**	0.92 (0.82–0.98)
PPV^§^	0.86 (0.74–0.93)	**0.93 (0.80–0.97)**	0.89 (0.76–0.96)
NPV^§^	0.81 (0.72–0.88)	**0.82 (0.73–0.88)**	0.77 (0.68–0.83)
AUC	0.90 (0.84–0.96)	**0.92 (0.86–0.98)**	0.88 (0.81–0.94)
**Clinical-radiological models (n = 71)**^**§§**^			
Accuracy^§^	**0.86 (0.76–0.93)**	0.83 (0.72–0.91)	0.79 (0.68–0.88)
OR^§^	**44.2 (10.4–187.0)**	27.1 (7.4–100.)	21.8 (5.5–85.5)
Sensitivity^§^	**0.82 (0.66–0.92)**	0.80 (0.63–0.91)	0.69 (0.52–0.83)
Specificity^§^	**0.91 (0.75–0.98)**	0.88 (0.71–0.965)	**0.91 (0.75–0.98)**
PPV^§^	**0.89 (0.73–0.96)**	0.85 (0.70–0.94)	0.87 (0.69–0.95)
NPV^§^	**0.85 (0.74–0.92)**	0.82 (0.71–0.90)	0.76 (0.67–0.84)
AUC	**0.93 (0.87–0.98)**	0.90 (0.83–0.97)	0.86 (0.78–0.95)

Regarding the teleradiologists’ performances, they correspond to conclusion = (1), (2), (3) or (4) versus conclusion = (5).

Accuracy, OR, sensitivity, specificity, PPV, NPV and AUC are given with 95% confidence interval.

The highest diagnostic performance measure for each line is highlighted in bold.

*AUC* area under the ROC curve, *CART* classification and regression tree; *NPV* negative predictive value; *OCTRs* on call teleradiologists; *OR* odds ratio; *PPV* positive predictive value; *Step-LR* stepwise binary logistic regression.

^§^The diagnostic performances are calculated after dichotomizing the predicted probability for RT-PCR + per 0.5.

^§§^Thirty of the 101 patients from the validation cohort were excluded because of missing values (all from clinical variables).

Regarding radiological models, the highest performances were reached with the CART model (AUC_validation_ = 0.91, 95%CI = [0.86–0.98] and accuracy_validation_ = 0.86, 95%CI = [0.78–0.92], versus AUC_validation_ = 0.90, 95%CI = [0.84–0.96] and accuracy_validation_ = 0.83, 95%CI = [0.74–0.90] in Step-LR model).

Regarding clinical-radiological models, the highest performances were reached with the Step-LR model (AUC_validation_ = 0.93, 95%CI = [0.87–0.98] and accuracy_validation_ = 0.86, 95%CI = [0.76–0.93], versus AUC_validation_ = 0.90, 95%CI = [0.83–0.97] and accuracy_validation_ = 0.83, 95%CI = [0.72–0.91] in CART model).

Regarding teleradiologists in real-life setting, in the whole study population and in the validation subcohort, the AUCs of the OCTRs’ conclusions (sub-categorized as (1–2–3) or (4) or (5)) were 0.89 (95%CI = [0.86–0.92]) and 0.88 (95%CI = [0.72–0.88]), respectively.

**Figure 4:**
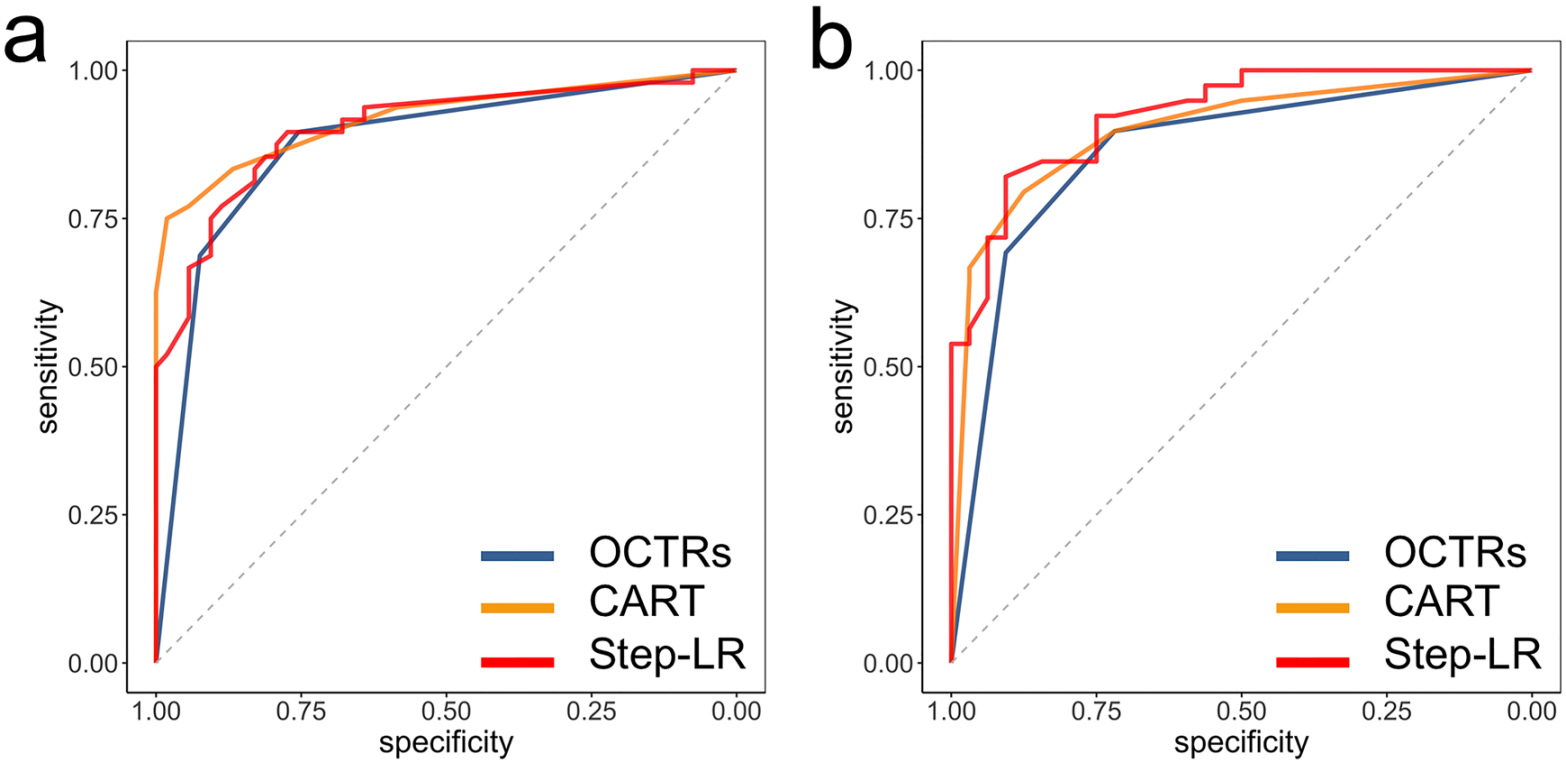
ROC curves of the final radiological (**a**) and clinical-radiological (**b**) multivariate models in the independent validation cohort. *CART* classification and regression tree; *Step-LR* stepwise binary logistic regression.

The ROC curves in the validation cohort are displayed on Fig. 4. None of the AUC_validations_ were significantly different (lowest p-value = 0.07 regarding the comparisons between the Step-LR clinical-radiological model and the OCTRs’ conclusions).

### Usefulness of the best model in case of indeterminate teleradiological conclusions

**Figure 5:**
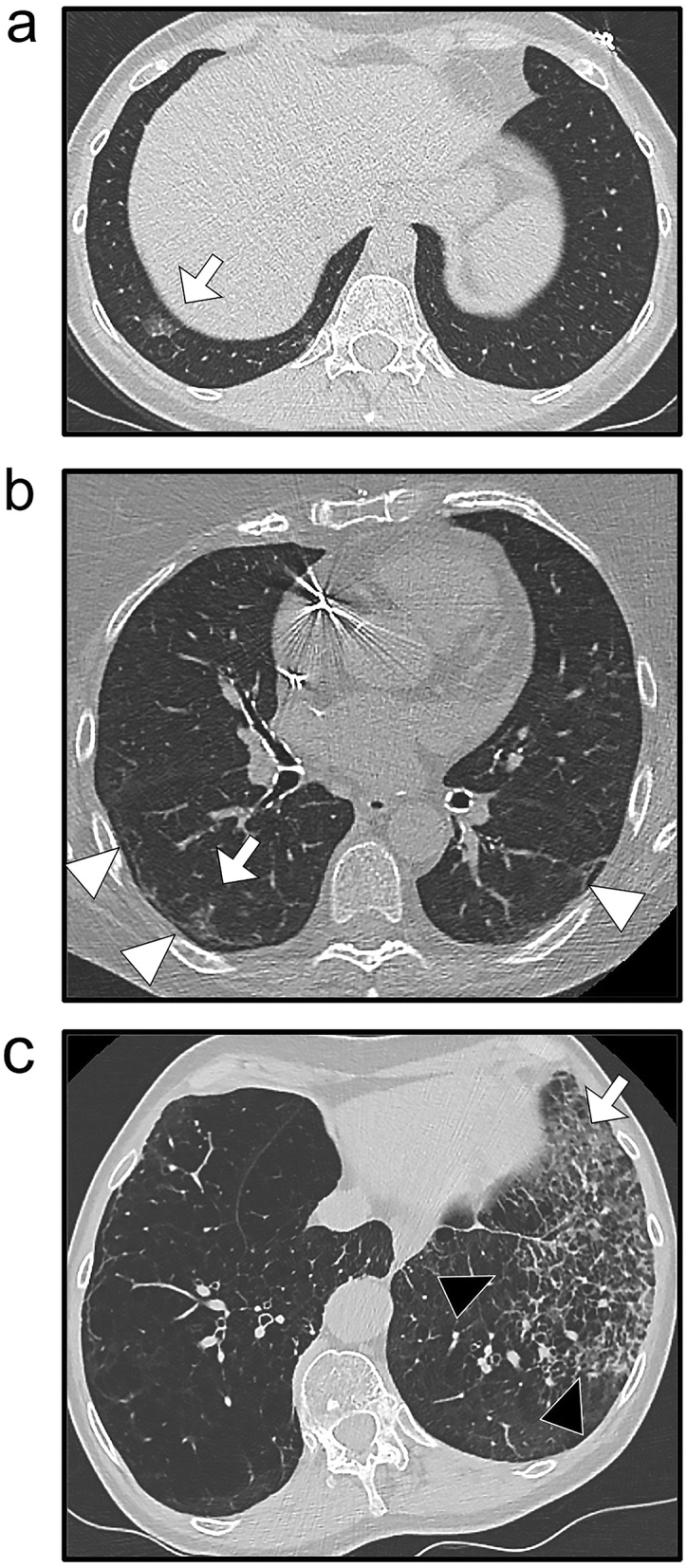
Added value of the final clinical-radiological model for patients with indeterminate/compatible radiological conclusions. (**a**) A 50-year-old woman presented at the emergency with chest pain, fever, and cough for less than one week. Chest CT showed a basal-predominant peripheral ground-glass opacity (GGO—white arrow) in the lower right lobe. The probability for RT-PCR + was 68.9%. (**b**) A 71-year-old woman presented at the emergency with cough, dyspnoea, fever and asthenia for 1–2 weeks. Chest CT showed bilateral peripheral fibrotic bands (white arrowheads) with a peripheral right GGO (white arrow). The probability for RT-PCR + was 65%. (**c**) A 61-year-old woman with a medical history of active smoking, emphysema and chronic obstructive pulmonary disease presented at the emergency with a cough, dyspnoea, fever and asthenia. Chest CT showed peripheral predominant intralobular reticulations in the lower left lobe (black arrowheads) with a single area of non-rounded GGO (white arrow). The probability for RT-PCR + was 57.9%. In the three cases, the RT-PCR was indeed positive.

In the subgroup of patients with an indeterminate conclusion (4), the probabilities for RT-PCR + with the clinical-radiological Step-LR model were significantly higher for patients with RT-PCR + than for patients with RT-PCR− (0.63 ± 0.28 versus 0.39 ± 0.27, respectively, p = 0.004). Figure 5 illustrates the potential application of this model for patients with an indeterminate/compatible conclusion. An excel macro is provided in Supplementary Data 4 so that the interested reader can test the Step-LR models.

### Misclassifications with the best model

Regarding the outliers of the model with the highest AUC (i.e., clinical-radiological step-LR), 3 out of the 32 patients (9.4%) with a negative RT-PCR in the validation cohort were classified positively by the model. Of these 3 patients, 2 were highly suspicious of COVID-19 and 1 was indeterminate according to the OCTRs. Conversely, 7 out of the 39 patients (17.9%) with a positive RT-PCR in the validation cohort were classified negatively by the model. Of these 7 patients, 4 had conclusions of (1), (2) or (3) according to the OCTRs.

### Clinical usefulness and decision curve analysis of the final models in the validation cohort

**Figure 6:**
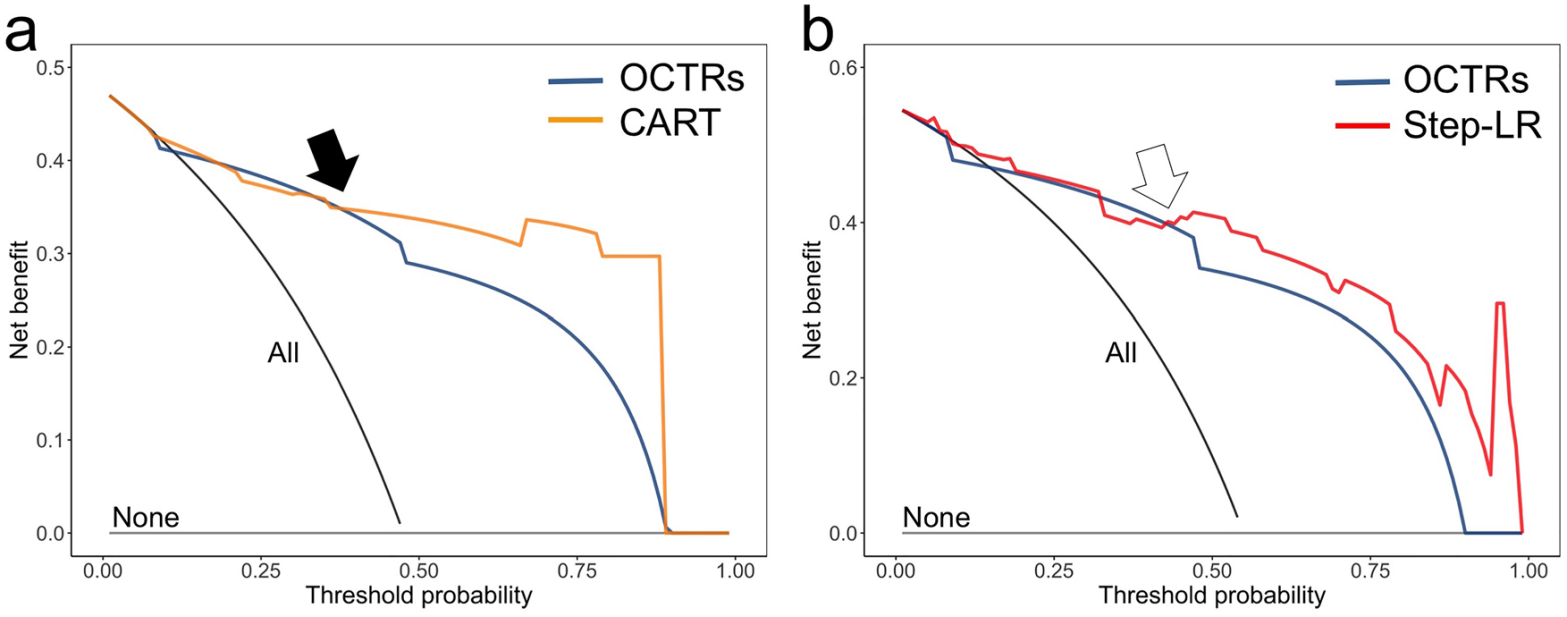
Decision curve analysis (DCA) of the final models and conclusions of the on-call teleradiologists (OCTRs) in the validation cohort, depending on data on which they were trained: (**a**) radiological and (**b**) clinical + radiological. The x-axis corresponds to the odds or risk threshold at which a patient would opt for COVID-19 management. The y-axis corresponds to the overall net benefit. The black line represents the ‘treat all’ patients COVID-19 strategy. The grey horizontal line represents the ‘treat none’ of the patients strategy. For both sources of data, the added value of the models over the ‘treat all’ strategy and the OCTRs readings are highlighted for risk thresholds above 0.43 (arrows). *CART* classification and regression tree, *Step-LR* stepwise logistic regression.

The DCAs showed that the OCTRs’ conclusion and the final models added more benefit than the ‘treat all approach’ above a risk threshold of approximately 0.05 (Fig. 6). The two final models added more net benefit than the ‘treat all’ strategy and the OCTRs’ conclusion for threshold probability above 0.43.

## Discussion

In this study, we developed practical and ready-to-use models to predict the RT-PCR status from categorical clinical and radiological variables that are routinely available or assessable by emergency physicians and radiologists without either expertise in thoracic imaging or computer science or additional blood samples. We purposely elaborated parsimonious models through a cautious variable selection including univariate filtering, assessment of correlations and stepwise selection. Our best models displayed high AUCs (> 0.90) and accuracy (> 0.85) on the external validation cohort, though they were not statistically different from those of OCTRs. Thus, these models could be helpful notably to weight indeterminate conclusions or to manage patients with a strong COVID-19 suspicion and negative (or lack of) RT-PCR^3^.

Our series represent a real-life multi-centric emergency population during the COVID-19 pandemic with well-balanced proportions of negative and positive RT-PCR results, making it appropriate for the development of predictive models. The clinical and radiological variables correlating with the RT-PCR status in the univariate analysis were consistent with the literature, namely, fever, asthenia, oxygen saturation, GGO, non-rounded consolidation, fibrotic bands, and intralobular reticulations with bilateral, diffuse, basal predominant and peripheral distributions^27–29^.

The aim of our models was to provide rapid assistance to non-specialized radiologists who may need support to conclude or modulate his report confidently without delaying patient management. We purposely chose classification algorithms that are easily explainable and do not require additional computing time, i.e., a binary logistic regression and a classification tree. These two algorithms are often used for benchmarking purposes in machine learning before using more complex models. In preliminary exploratory analyses, we actually tested other algorithms such as random forests or categorical boosted trees, which indeed showed slightly higher AUCs but could not be visually represented and explained to radiologists. Interestingly, similar performances of our models in the training and validation cohorts highlight the lack of overfitting and their good generalizability to new patients.

Improving COVID-19 diagnosis with deep learning frameworks has already been attempted with good results, but without remarkable added value compared with the diagnostic performances of traditional radiological assessment, OCTRs or our models as calculated in the independent validation set. Indeed, previous studies have found that the sensitivity, specificity and accuracy of radiologists on chest CT were 0.97, 0.56 and 0.72, respectively^30^. Using a quantitative assessment of the main COVID-19 radiological features (through volumes of GGO and fibrotic alterations) did not increase sensitivity and specificity (0.68 and 0.59 with GGO, respectively, and 0.86 and 0.44 with fibrotic alterations, respectively)^31^. The AUCs of these deep learning models mostly ranged between 0.70 and 0.95, while sensitivity and specificity, when given, ranged between 0.84 and 1, and 0.25 and 0.96, respectively^15–20^.

Other studies proposed practical scores such as the PSC-19, which relies on four variables (history of exposure, leukocyte count, peripheral lesion and crazy paving patterns), with AUC = 0.91, sensitivity = 0.88 and higher specificity of 0.92^22^. However, this score requires a blood sample and could be of limited use when investigating a patient in a new COVID-19 cluster of patients without proven exposure. Chen et al. also combined an explainable machine learning algorithm (i.e., penalized logistic regression) and bio-clinical-radiological variables^21^. They built three models: bio-clinical alone, radiological alone and bio-clinical-radiological models. Surprisingly, the results of the final models in the validation cohort showed the highest performances when radiological variables were not taken into account (AUCs = 0.97, 0.81 and 0.94, respectively). Ridge and/or LASSO penalized logistic regressions have also been investigated in our preliminary data exploration but did not show added value to Step-LR. While deep learning studies trained their models in large cohorts of hundreds of patients, it should be noted that these two practical studies did not exceed one hundred patients, questioning their validity. In addition, the performances of standardized radiological conclusions were missing in all these studies involving artificial intelligence. It should be noted that alternative measures could have been used to evaluate and compare classification models, instead of the AUC. We purposely chose the AUC because it was the most frequently used estimators in similar prior studies and it is adapted to well-balanced study population regarding the outcome.

Our results also highlight the very good performances of radiologists in daily practice, which can be explained by the considerable increase in knowledge of the COVID-19 radiological presentations since the first peer-reviewed studies in January–February 2020, attained through open-source educational publications, webinars and recommendations issued by national and international radiological societies, which were immediately relayed to the teleradiologists working at IMADIS. Indeed, comparisons of AUCs between best models and OCTRs’ conclusions did not reach significance, which could have been due to lack of power. Additionally, OCTRs could ask for their colleagues’ advice when facing a complicated case, as they were never alone in one of our two emergency interpretation centres during on-call duty. Therefore, though our predictive models showed comparable performances with other machine- and deep-learning models, and slightly higher performances than OCTRs’ conclusions, the gain was not striking, leading to no significant difference according to the Delong test. Few studies have focused on the specificity of teleradiologists compared with conventional radiologists. It should be noted that the organization of teleradiology could greatly vary from one group to one another. IMADIS has the particularity to gather teams of teleradiologists in interpretation centers, with the constant ability to interact with colleagues and solicit help. Banaste et al*.* have demonstrated that IMADIS structuration enables to organize systematic second readings of whole body CT scanners in multiple traumas patients, with good diagnostic performances of OCTRs whatever the amount of activity and the hours of the day^32^. Regarding the OCTRs’ accuracy for diagnosing COVID-19, Nivet et al. have also shown that implementing the semi-quantitative assessment as proposed by SFR was feasible and provided high sensitivity (0.92), specificity (0.75–0.84), PPV (0.77–0.84) and NPV (0.91–0.92) depending on the reading and when considering the indeterminate/compatible group as positive^12^.

To illustrate eventual clinical applications of the final models, we used DCAs, which are a popular alternative to cost-effectiveness studies^24^. Regarding the two settings (radiological data alone and clinical-radiological data), we found similar shapes of the DCAs for the OCTRs’ conclusion and the final models. Interestingly, at worst, the net benefits of the models were equivalent to those of OCTRs (for the intermediate risk threshold) and to the ‘treat all’ strategy (for the very low risk threshold). However, the machine learning models would be complementary to the OCTRs’ conclusion and would improve the net benefit for patients from intermediate- to high-risk thresholds. Practically, we believe that our models could be useful for patients with (i) high suspicion but non-available RT-PCR status or negative first RT-PCR, and (ii) indeterminate OCTR’s conclusion. A high probability per our models would lead to the isolation of the patients and the achievement of another RT-PCR.

The measurements of performance in our models should also be considered with the disease prevalence during our period of inclusion (≈ 48%) and the nature of the study population^33^. Indeed, although PPV and NPV are useful to rapidly sort patients with COVID-19 suspicion, they depend on this prevalence, which fluctuates depending on the country and public health measures.

Our study has certain limitations. First, the RT-PCR is an imperfect gold standard, which may explain why no study has ever reached perfect performance. Indeed, the analysis of the outliers of the best model in the validation cohort with fully available clinical-radiological variables (n = 10, 3 false positives and 7 false negatives) demonstrated that 6 of these patients either could have performed their chest CT before the disease manifestation or were false negative by RT-PCR. This finding stresses the risk of false negatives with chest CT due to the delay between the beginning of clinical symptoms and the appearance of the COVID-19 semiology on chest CT. Second, approximately 25–33% of clinical data were missing because they were collected in real-life emergency situations. Third, some radiological features were not evaluated because they were published after the beginning of the COVID-19 IMADIS workflow (for instance: multifocality and thickened vessels). Fourth, a history of exposure was rarely collected in our cohort although it could have been an important predictor. Fifth, biological markers were not available because they were rarely dosed before asking for the chest CT. Sixth, although the OCTRs’ conclusions were prospectively collected, the radiological features that were necessary to elaborate the predictive models were not collected in emergency situations, which could have led to the overestimation of the associations of these features with the RT-PCR status. Seventh, it is worth noting that our study did not propose specific models to differentiate non-COVID-19 infectious lung diseases from COVID-19. Though an important corollary question, this was not our first aim. Indeed, our population represents a real-life emergency cohort and some patients could have had non-infectious diseases (: conclusion (2)), or non-pulmonary infections for instance (: conclusion (1)), in addition to non-COVID-19 lung infectious diseases (: conclusion (3)). Finally, although widely used and significantly reproducible over multiple raters, the radiological features were qualitatively or semi-quantitatively assessed, which introduces a risk of subjectivity and could bias the performances of the model in other validation cohorts^12^.

To conclude, we presented one of the largest French multicentric emergency cohort including prospective standardized reports following national recommendations. Our findings illustrate the high diagnostic performances of the OCTRs working in a teleradiological structure entirely dedicated to emergency imaging, which promoted continuous training and collaborative work. This setting enables us to propose free and practical models built on easily available clinical and radiological data provided by emergency physicians and OCTRs. These models provide a probability for positive RT-PCR, which could be used by general radiologists in case of indeterminate radiological conclusions, and no/limited availability to RT-PCR in order to confidently conclude their reports in daily practice.

## Supplementary Information

Supplementary Information 1.

Supplementary Information 2.

## Data Availability

The datasets analyzed during the current study will be publicly available (Being submitted to Springer Nature Database). Details regarding the statistical analysis can be made available from the corresponding author on reasonable request.

## Abbreviations

95%CI: 95% Confidence interval
AUC: Area under the receiver operating characteristic curves
CART: Classification and regression tree
COVID-19: Coronavirus disease 2019
CT: Computed tomography
DCA: Decision curve analysis
GGO: Ground-glass opacities
NPV: Negative predictive value
OCTR: On-call teleradiologists
PPV: Positive predictive value
RT-PCR: Real-time reverse transcription polymerase chain reaction
SARS-CoV-2: Severe acute respiratory syndrome coronavirus 2
SFR: French society of radiology
Step-LR: Stepwise binary logistic regression
