# Supplementary material for: Practical clinical and radiological models to diagnose COVID-19 based on a multicentric teleradiological emergency chest CT cohort
Source: Sci Rep. 2021 Apr 26;11:8994. doi: 10.1038/s41598-021-88053-6 (PMC8076229; doi:10.1038/s41598-021-88053-6)
Supplement: Supplementary file 1 — Supplementary Information 1. [file 41598_2021_88053_MOESM1_ESM.docx]

**SUPPLEMENTAL DATA**

**Supplemental Data 1.** Partner emergency departments involved in the prospective recruitment of patients.

| **Hospital** | **No. of patients** |
| --- | --- |
| A | 15/513 (2.9%) |
| B | 24/513 (4.7%) |
|  |  |
| C | 19/513 (3.7%) |
| D | 52/513 (10.1%) |
| E | 23/513 (4.5%) |
| F | 31/513 (6%) |
| G | 40/513 (7.8%) |
| H | 1/513 (0.2%) |
| I | 25/513 (4.9%) |
| J | 151/513 (29.4%) |
| K | 103/513 (20.1%) |
| L | 1/513 (0.2%) |
| M | 2/513 (0.4%) |
| N | 12/513 (2.3%) |
| O | 14/513 (2.7%) |

NOTE. – Data refer to numbers of patients with percentage in parentheses.

**Supplemental Data 2.** Radiological variables assessed in the study. Conclusions were adapted from the French Society of Radiology proposals, as follows: (1) normal chest CT; (2) lung abnormalities but non-consistent with a pulmonary infection (herein, cardiogenic pulmonary edema); (3) consistent with a lung infection but not with COVID-19 (herein, acute community-acquired pneumonia); (4) indeterminate, however compatible with COVID-19; (5) strongly suspicious of COVID-19 (bilateral peripheral basal-predominant GGO). Abbreviations: GGO : ground-glass opacities


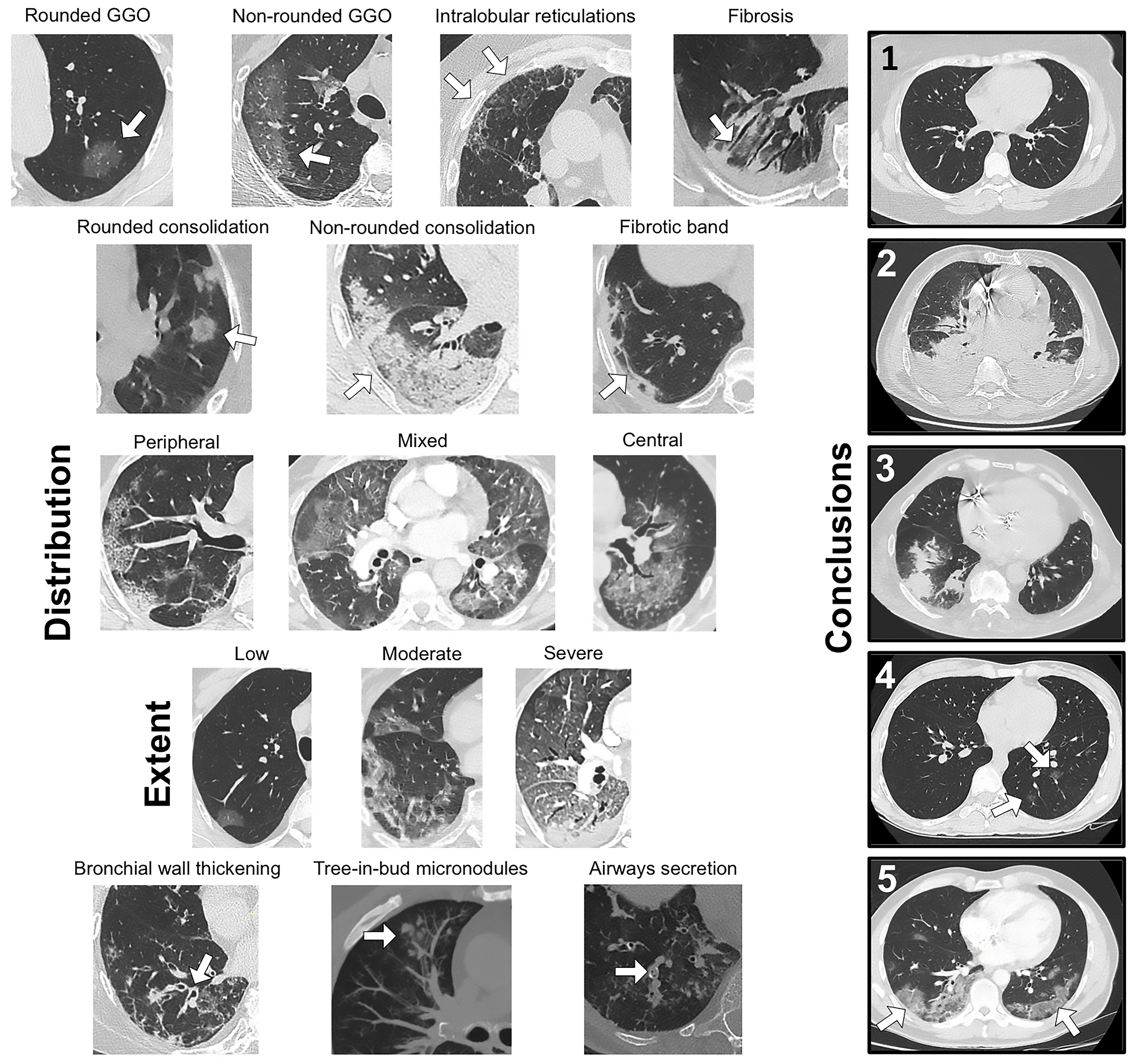


**Supplemental Data 3.** Final models performances on the training cohort

| **Performance measures** | **Models** | |
| --- | --- | --- |
|  | **Step-LR** | **CART** |
| **Radiological models (n = 412)** | |  |
| **Accuracy^§^** | 0.88 (0.84 - 0.91) | 0.87 (0.83 - 0.90) |
| **OR^§^** | 52.7 (29.1 - 95.3) | 43.8 (24.7 - 77.7) |
| **Se^§^** | 0.88 (0.83 - 0.92) | 0.86 (0.81 - 0.91) |
| **Sp^§^** | 0.87 (0.82 - 0.92) | 0.87 (0.82 - 0.92) |
| **VPP^§^** | 0.87 (0.82 - 0.90) | 0.86 (0.81 - 0.9) |
| **VPN^§^** | 0.89 (0.85 - 0.92) | 0.87 (0.83 - 0.91) |
| **AUC** | 0.92 (0.89 - 0.95) | 0.90 (0.87 - 0.93) |
| **Clinical-radiological models (n = 295)^§§^** | |  |
| **Accuracy^§^** | 0.91 (0.87 - 0.94) | 0.87 (0.83 - 0.91) |
| **OR^§^** | 107. (47.8 - 239.3) | 48.9 (24.5 - 97.5) |
| **Se^§^** | 0.91 (0.85 - 0.95) | 0.86 (0.79 - 0.91) |
| **Sp^§^** | 0.91 (0.86 - 0.95) | 0.89 (0.83 - 0.93) |
| **VPP^§^** | 0.91 (0.85 - 0.94) | 0.87 (0.82 - 0.92) |
| **VPN^§^** | 0.92 (0.87 - 0.95) | 0.88 (0.82 - 0.91) |

NOTE. – ^§^. The diagnostic performance measures were calculated after dichotomizing the predicted probability for RT-PCR+ per 0.5 (i.e. < 0.5 corresponds to predicted RT-PCR- and > 0.5 to predicted RT-PCR+).

^§§^. 118 of the 413 (28.6%) patients from the validation cohort were excluded because of missing values (all from clinical variables).

Accuracy, OR, sensitivity, specificity, PPV, NPV and AUROC are given with 95% confidence interval.

The highest diagnostic performance measure for each line is highlighted in bold.

Abbreviations: AUC: area under the ROC curve, CART: classification and regression tree; NPV: negative predictive value; OR: odds ratio; PPV: positive predictive value; Step-LR: stepwise binary logistic regression.
